# Study on preparation of chickpea peptide and its effect on blood glucose

**DOI:** 10.3389/fnut.2022.988628

**Published:** 2022-09-16

**Authors:** Xuemei Ma, Xing Fan, Deping Wang, Xianai Li, Xiaoyun Wang, Jiangyong Yang, Chenggong Qiu, Xiaolu Liu, Guangxian Pang, Redili Abra, Liang Wang

**Affiliations:** ^1^College Life Science and Technology, Xinjiang University, Urumqi, China; ^2^Xinjiang Arman Food Group Co. LTD, Urumqi, China; ^3^Shenxin Science and Technology Cooperation Base Co. LTD, Urumqi, China

**Keywords:** chickpea, fermentation strains, peptide, α-glucosidase, molecular weight

## Abstract

Chickpeas are the third largest bean in the world and are rich in protein. In this study, chickpea peptides were prepared by the enzyme-bacteria synergy method. Taking the peptide yield as the index, we first screened 8 strains suitable for the fermentation of chickpea peptides from 16 strains, carried out sodium dodecyl sulfate polyacrylamide gel electrophoresis, and then screened 4 strains with the best decomposition effect of chickpea protein. The molecular weight, amino acid content, and α-glucosidase inhibitory activity of the chickpea peptides fermented by these four strains were detected. Finally, the strains with the best α-glucosidase inhibitory activity were obtained, and the inhibitory activities of the different molecular weight components of the chickpea peptides fermented by the strains with the best α-glucosidase inhibitory were detected. It was found that *Bifidobacterium species* had the best fermentation effect, and the highest peptide yield was 52.99 ± 0.88%. *Lactobacillus thermophilus* had the worst fermentation effect, and the highest peptide yield was 43.22 ± 0.47%. Sodium dodecyl sulfate-polyacrylamide gel electrophoresis (SDS-PAGE) showed that *Bifidobacterium species, Lactobacillus acidophilus, Lactobacillus rhamnosus*, and *Lactobacillus paracasei* have a better effect on the decomposition of chickpea protein in the fermentation process, and the molecular weight of their fermented peptides is basically below 20 KDa. Among the four strains, the α-glycosidase inhibition of chickpea peptide fermented by *Lactobacillus acidophilus* was the best, which was 58.22 ± 1.10% when the peptide concentration was 5.0 mg/ml. In chickpea peptide fermented by *Lactobacillus acidophilus*, the influence of molecular weight on the inhibitory activity is not obvious when the molecular weight is <10 kD, and the molecular weight range of the best inhibitory effect is 3–10 kD, and the inhibitory rate of α-glucosidase is 37 ± 1.32% at 2.0 mg/ml. This study provides a theoretical basis for the study of a new preparation method for chickpea peptide and its hypoglycemic effect.

## Introduction

Chickpea (Cicer arietinum L.), also known as Bengal bean, is an annual plant (1) in the legume family, accounting for 16% of the global legume production (FAO (2020), FAOSTAT statistical database). It is widely believed that chickpea is nutritious and it contains a variety of beneficial and rich compounds, such as carbohydrates, proteins, unsaturated fatty acids, minerals, vitamins, dietary fiber, and a series of isoflavones (2). Chickpea contains 15–25% protein and is considered a good source of carbohydrates and protein, which is generally considered to be superior to other beans (3). Chickpea contains 18 kinds of amino acids (2), 8 of which are essential. Sodium dodecyl sulfate-polyacrylamide gel electrophoresis (SDS-PAGE) was used to analyze different types of proteins in chickpea seeds, and the results showed that six kinds of chickpea seeds mainly contained albumin (4), globulin, gliadin, and salt-soluble protein.

Protein hydrolysates are potential sources of peptides with various bioactive properties. Several processes have been developed to produce peptides, such as biological and chemical methods. Microbial fermentation of food proteins to produce peptides is an alternative approach to enzymatic and chemical peptide production. As peptides produced by food fermentation have good health effects, they can solve a variety of health diseases (5), and the fermentation process has attracted extensive attention because of its role in improving functional characteristics and reducing anti-nutritional factors (6–8). Some studies have found that fermentation can increase the value of beans by increasing vitamin and mineral content, increasing carbohydrate bioavailability (9), and increasing the production of free polyphenols and bioactive peptides. More significantly, fermentation using certain beneficial strains has been shown to produce bioactive peptides capable of preventing certain diseases (10). Chickpeas have been evaluated for their health benefits. Chickpea peptides can be produced by isolating proteins from raw or processed chickpeas and subsequently hydrolyzing proteins using enzymes or acid/base methods. Various proteins and peptides in chickpea and chickpea processed extracts have antibacterial effects (11), hemagglutination activity, antioxidant and anti-tumor effects (12), and hypoglycemic (13) and lipid-lowering effects (14). It is a pity that the beneficial effects of chickpea fermenting peptides on lowering blood glucose are still relatively little studied, and their anti-diabetic potential has not been widely evaluated.

It is estimated that the number of people with type 2 diabetes (T2D) will increase by more than 50% between 2017 and 2045. The global healthcare cost of controlling and treating T2D is approximately $850 billion per year (15). To treat T2D, it is necessary to reduce the rate of glucose uptake in the small intestine, which can reduce the postprandial rise in gut hormones and insulin. In humans, most dietary carbohydrates are digested and converted to glucose by α-glucosidase and α-amylase. In the case of glucose-lowering peptides, peptides can exert their activities through different metabolic pathways. A notable example is the inhibition of α-glucosidase. This enzyme is responsible for breaking down the polysaccharide into glucose, which can be absorbed. If the patient suffers from diabetes, the blood glucose level will rise and have adverse effects on the body (16). The inhibitory level of α-glucosidase in chickpea peptide was significantly higher than that in lentils or quinoa. The difference between the different sources can be explained by the amino acid content of each protein, as chickpea has a high content of arginine and lysine (17, 18), which in the peptide will significantly increase the inhibitory activity of α-glucosidase.

In this article, the yield of chickpea peptide produced by the fermentation of 16 different strains was studied by the combination of enzyme bacteria. Eight strains of the best fermentation were screened by the yield of peptide and confirmed by the Kjeldahl method. The peptides obtained by the fermentation of these 8 strains were subjected to sodium dodecyl sulfate polyacrylamide gel electrophoresis (SDS-PAGE) and four strains with the best effect of decomposing chickpea protein were obtained. The molecular weight, amino acid content, and α-glucosidase inhibitory activity of the fermented chickpea peptides obtained from the above four strains were detected. Finally, the chickpea peptides with the highest α-glucosidase inhibitory activity were obtained, as well as the inhibitory activity of its different molecular weight components was also explored.

## Materials and methods

### Materials and instruments

The chickpeas were from Xinjiang, China and all chemical reagents were analytically pure. The water bath pot and incubator were purchased from Beijing Yongguangming Medical Instrument Co., LTD., China. Uv-visible spectrophotometer was purchased from Shanghai Mpoda Instrument Co., LTD., China. The pulverizer was purchased from Guangdong Fangtai Electric Co., LTD., China. The centrifuges were purchased from Jiangsu Jinyi Instrument Technology Co., LTD., China. The electrophoresis apparatus was purchased from Beijing Liuyi Instrument Factory. Microscope was from Motic. And whirlpool mixer was purchased from Shanghai Youbuy Automation Equipment Co., LTD., 80 mesh screen purchased from Shaoxing Shangyu Zhangxing yarn screen factory. A-amylase (3,800 U/g) was obtained from Beijing Aobo Star Biotechnology Co., LTD and lipase (100,000 U/g) was obtained from Shandong Longkete Enzyme Preparation Co., LTD.

### Methods

#### Preparation of chickpea peptide

The dried chickpeas were crushed by a shredder and sifted through 80 mesh. The ratio of material to water was 1:20 (W/V), and the mixture was precooked with water at 85 °C for 30 min and then placed at room temperature. The reaction was terminated by adding 0.5% α-amylase and 0.1% lipase based on the weight of chickpea flour in a water bath at 50 °C for 6 h and then inactivating the enzyme at 85 °C for 30 min. After the enzyme was killed, the chickpea solution was put to room temperature, and 16 strains were added, respectively according to the initial bacterial density of 1 × 10^7^ CFU/ml. The chickpea solution after inoculation was static fermented at 37 °C, and the peptide content and the total number of colonies were detected every 8 h until the peptide content tended to be stable or decreased. The final fermentation time was determined to be 40 h according to the pre-experiment.

#### Determination of peptide content

In this study, we took 1 ml of sample solution and added 1 ml of 15% (W/W) trichloroacetic acid (TCA) aqueous solution. It was mixed and allowed to stand for 10 min, and then centrifuged at 4,000 r/min for 10 min. Then, we took 1 ml of supernatant and added 4 ml of biuret reagent to it. Again, the solution was mixed and allowed to stand for 30 min. At the same time, l ml of water and 4 ml of biuret reagent were mixed and stood as blanks, and the absorbance value was measured at 540 nm. The peptide concentration C (mg/ml) in the hydrolysate was calculated by the standard regression equation, and the yield of peptide was the percentage of soluble peptide obtained by fermentation hydrolysis in total protein.

#### Detection of nitrogen content in fermentation residue

To prove the accuracy of peptide yield data, the fermentation residue of each strain at the optimal fermentation time was dried and the nitrogen content was detected according to the Kjeldahl method GB5009.5-2016. The hydrolysis degree of chickpea fermented by different strains was calculated by taking the water loss of fermentation residue as 80% and the protein conversion coefficient as 6.25.


$$\begin{array}{l} \text{Degree of hydrolysis }(\%)\;=\;(\text{Protein content in raw materials}\\ -\text{ Protein content in the residue})/\\ \text{Protein content in raw materials }\times \;100. \end{array}$$

#### Sodium dodecyl sulfate-polyacrylamide gel electrophoresis

To further screening the fermentation effects of better strains, Sodium dodecyl sulfate polyacrylamide gel electrophoresis (SDS-PAGE) was performed on chickpea peptides fermented by 8 strains selected with good fermentation effect based on peptide yield. Explore the approximate distribution of peptide molecular weight of each strain after fermentation to further screen the strains suitable for the fermentation of chickpea.

(1) Sample processing: protein samples and molecular weight standard proteins were denatured before loading electrophoresis. Usually, the sample protein solution was mixed with an equal volume of sample buffer and placed in an Eppendorf tube. The mixture was heated at 95–100 °C for 5 min, immediately placed on ice, or stored at −20 °C for re-analysis.
(2) Sample loading: after the sample was prepared, the sample loading could be electrophoresed.

First the sample comb was removed and each sample well was washed with double steaming water. Then the electrophoresis buffer was added to the sample well and the electrophoresis tank was also filled with the electrophoresis buffer. After treatment, add samples for electrophoresis according to the needs of the experiment.

(3) Electrophoresis: electrophoresis was usually carried out by steady current. The current was generally 18 mA, and the time was about 3–4 h.

#### Molecular weight detection

(1) The main operating parameters of the instrument: column: TSKgel 2000 SWXL 300 mm × 7.8 mm; mobile phase: acetonitrile/water/trifluoroacetic acid, 45/55/0.1 (V/V); detection: UV 220 nm; flow rate: 0.5 ml/min; column temperature: 30 °C.
(2) Sample preparation: weigh 100 mg of the sample into a 10 ml volumetric flask, dilute it with mobile phase to the scale, and filter it with 0.45 μm microporous filter membrane for sample injection.
(3) The sample solution was analyzed under the above chromatographic conditions, and the data were processed with GPC software to obtain the peptide phase in the sample, its molecular mass distribution, and its distribution range.

#### Detection of the amino acid content

Amino acid composition was determined by using acid hydrolysis, derivatization, and high-performance liquid chromatography (HPLC). Tryptophan was determined by alkaline hydrolysis according to the Association of Official Analytical Chemists (AOAC) method. The results were expressed in mg/ml of dry matter.

#### Detection of α-glucosidase inhibition rate

Refer to the determination method of Boath et al. (8), Raju et al. (19), and slightly modify it. Weigh an appropriate amount of α-glucosidase and α- D-glucopyranoside (PNPG) were prepared into 20 u/ml and 3 mmol/ml solutions with phosphate buffer (PBS, 0.2 M, pH 6.8). Accurately suck 50 ul of PBS, 50 ul of PNPG solution, and 50 ul of enzymatic hydrolysate solution with a pipette gun into 96 well enzyme label plate, incubate at 37 °C for 10 min, and then add 100 ul of α-incubate with glucosidase solution for 30 min, and finally add 150 ul of Na_2_CO_3_ solution (1 mol/L) to terminate the reaction, and measure the absorbance value at 405 nm wavelength and record it.

The calculation formula of inhibition rate is as follows:


$$\begin{array}{l} \;\alpha -\text{glucosidase inhibition rate }\left(\%\right)\\ =\;\left[1-\left({\text{A}}_{\text{a}}-{\text{A}}_{\text{b}}\right)/\left({\text{A}}_{\text{c}}-{\text{A}}_{\text{d}}\right)\right]\;\times 100 \end{array}$$

Where, A_a_ is the absorbance of the sample, A_b_ is the absorbance of the sample control group, A_c_ is the absorbance of the blank group, and A_d_ is the absorbance of the blank control group.

#### Hierarchical treatment of chickpea peptide

The supernatant of chickpea fermentation peptide was taken and centrifuged successively with the interception molecular weight of 1, 3, and 10 kD ultrafiltration centrifuge tube (MilliIPore) to obtain the peptide solution with molecular weight of <1, 1–3, 3–10, and >10 kD, which was collected and stored at −4 °C for future use.

#### Data analysis

All data are presented as the mean ± standard deviation (SD) of at least three independent experiments. Statistical significance (*p* < 0.05; Duncan's test) was determined using SPSS version 20.0 software (IBM Corp., Armonk, NY). Graphs were made using Origin 2021 software (OriginLab Corporation, Northampton, MA, USA).

## Results and analysis

### Analysis of yield of chickpea peptide by single strain fermentation

**Figure 1:**
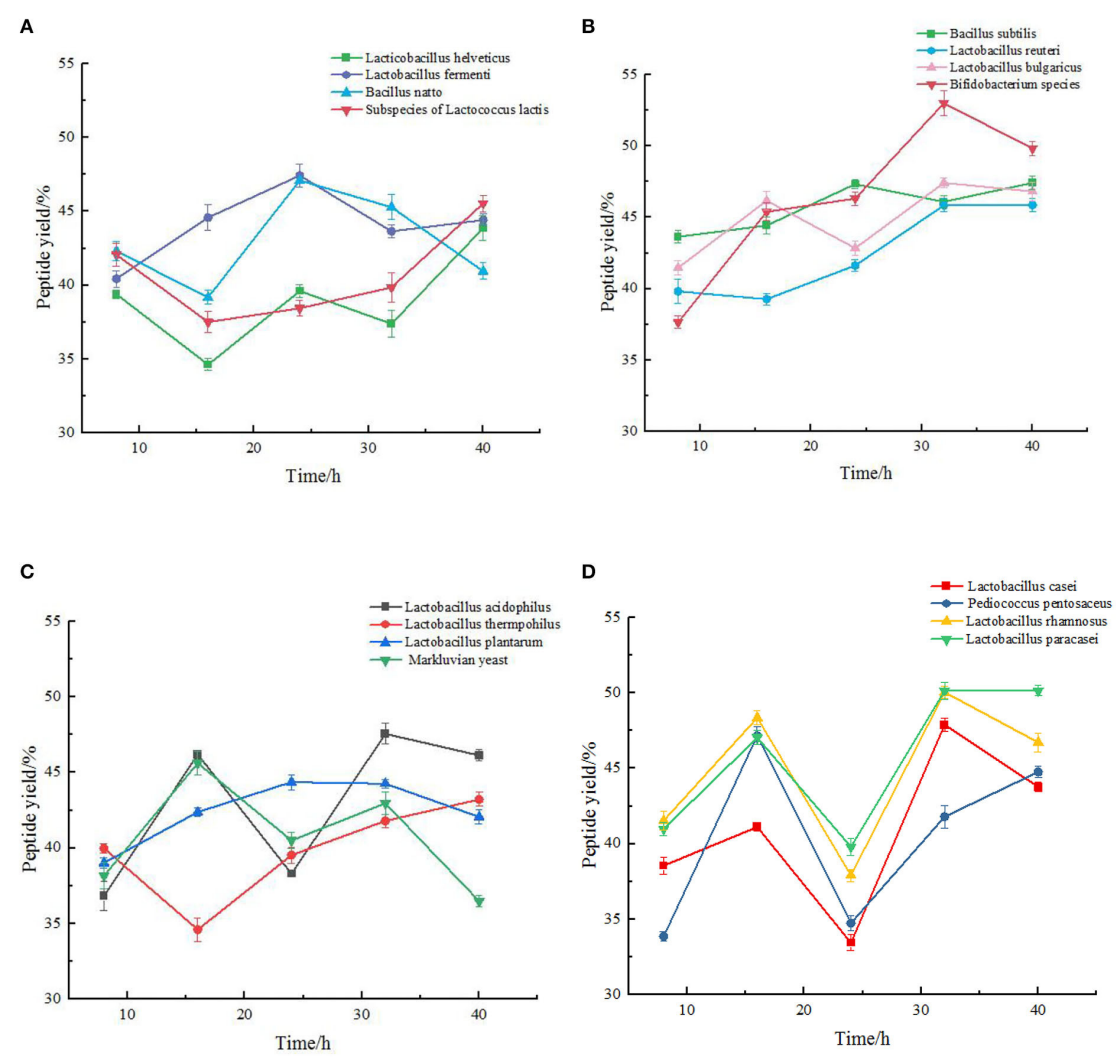
Change of peptide yield (%) during the fermentation of chickpea by different species. The values are presented as the mean of three replicates 1 standard deviation (SD). **(A)** Variation of peptide yield of chickpea fermented by *Lactobacillus helveticus, Lactobacillus fermenti, Bacillus natto*, Subspecies of *Lactococcus lactis* respectively. **(B)** Variation of peptide yield of chickpea fermented by *Bacillus subtilis, Lactobacillus reuteri, Lactobacillus bulgaricus, Bifidobacterium* species respectively. **(C)** Variation of peptide yield of chickpea fermented by *Lactobacillus acidophilus, Lactobacillus thermophilus, Lactobacillus plantarum*, and *Maxruvius yeast* respectively. **(D)** Variation of peptide yield of chickpea fermented by *Lactobacillus casei, Pediococcus pentosaceus, Lactobacillus rhamnosus*, and *Lactobacillus paracasei* respectively.

It can be seen from Figure 1 that among the 16 strains, the yield of peptide produced by most of them fluctuated continuously during the fermentation process, which may be due to the continuous condensation and dispersion of small molecular peptides and amino acids in this process. In the four figures, the peptide yield of chickpea fermented by most strains reached the peak at approximately 32 h, and then stabilized or decreased. The *Bifidobacterium species* had the best fermentation effect, and the highest peptide yield was 52.99 ± 0.88%. *Lactobacillus thermophilus* had the worst fermentation effect, and the highest peptide yield was 43.22 ± 0.47%. As shown in Figure 1A, the fermentation trend of the four strains was not very consistent. The peptide of *Lactobacillus helveticus* and *Lactobacillus fermenti* increased first and then decreased during the fermentation process, and gradually decreased after reaching the peak in about 24 h. The peptide yield from *Bacillus natto* and *subspecies of Lactococcus lactis* almost showed a slowly increasing trend during fermentation. Figure 1B showed the trend of peptide yield in the fermentation process of *Bacillus subtilis, Lactobacillus reuteri, Lactobacillus bulgaricus*, and *Bifidobacterium species*. We can clearly see that the fermentation trend of the four strains is almost the same, and their indicators reach the maximum at 32 h of fermentation, and gradually decline or become gentle in the following time. The fermentation trends of the four strains (*Lactobacillus acidophilus, Lactobacillus thermophilus, Lactobacillus plantarum*, and *Maxruvius yeast*) shown in Figure 1C were also roughly the same. However, it can be clearly seen that during the fermentation of chickpea by *Lactobacillus thermophilus*, the peptide yield was the lowest at 16 h. Taking this time point as the inflection point, the peptide yield gradually increased in the subsequent fermentation. The fermentation trends of the four strains (*Lactobacillus cas*ei, *Pediococcus pentosaceus, Lactobacillus rhamnosus*, and *Lactobacillus paracasei*) in Figure 1D were basically the same. The peptide yield of chickpea fermented by these four strains reached the first small peak at 16 h, and tended to be flat or decline after reaching the peak at 32 h. According to Figure 1, 8 strains (*Bifidobacterium species, Lactobacillus acidophilus, Lactobacillus rhamnosus, Lactobacillus paracasei, Lactobacillus fermenti, Lacillus subtilis, Lactobacillus bulgaricus*, and *Lactobacillus casei*) with the best peptide yield can be preliminarily screened, and the optimal fermentation time is determined to be 32 h.

### Detection results of the protein content in the fermentation residue

**Figure 2:**
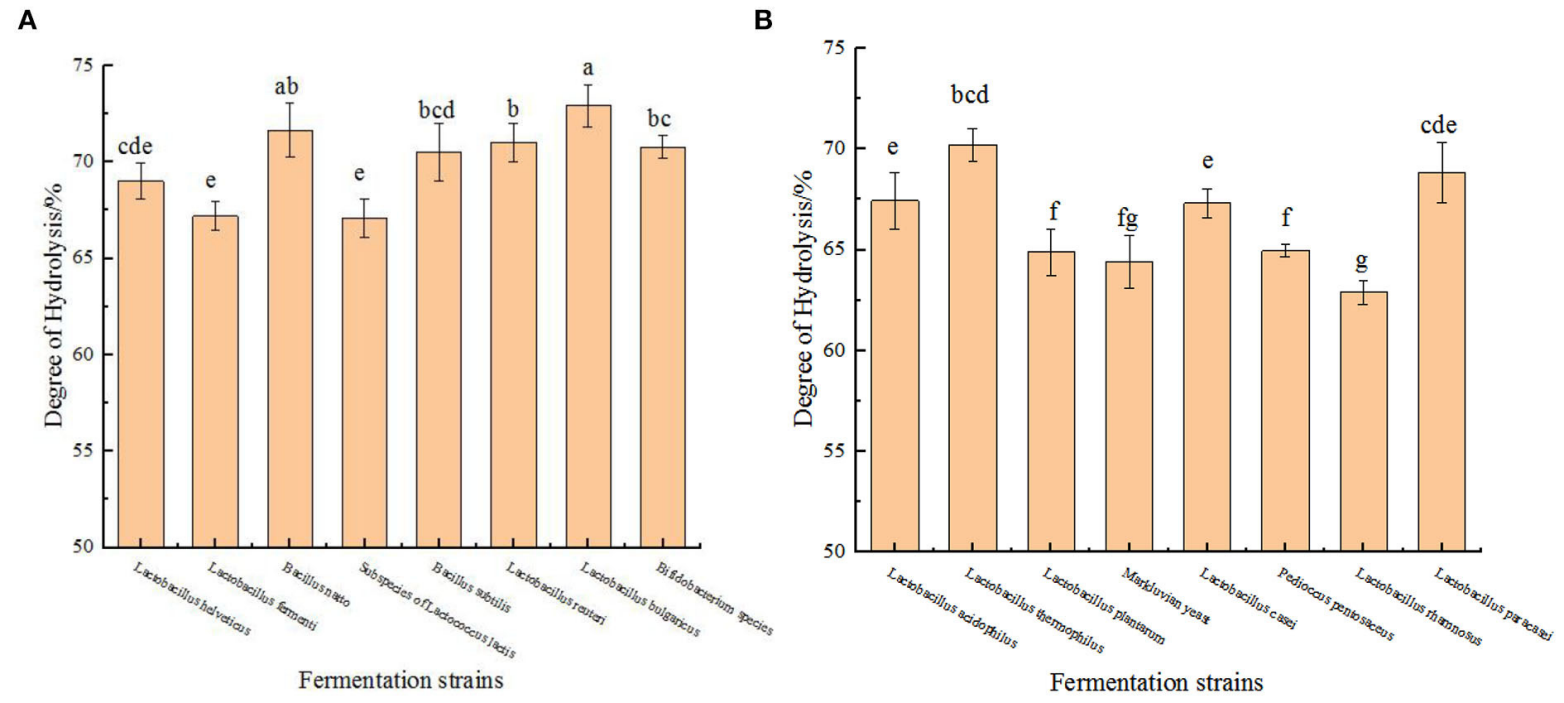
Detection results of the protein content in the fermentation residue. **(A)** Hydrolysis degree produced by fermentation of chickpea by 8 strains (*Lactobacillus helveticus, Lactobacillus fermenti, Bacillus natto, Subspecies of Lactococcus lactis, Bacillus subtilis, Lactobacillus reuteri, Lactobacillus bulgaricus, Bifidobacterium* species) at their respective optimal fermentation time. **(B)** Hydrolysis degree produced by fermentation of chickpea by 8 strains (*Lactobacillus acidophilus, Lactobacillus thermophilus, Lactobacillus plantarum, Markluvian yeast, Lactobacillus casei, Pedioccus pentosaceus, Lactobacillus rhamnosus, Lactobacillus paracasei*) at their respective optimal fermentation time. The values are presented as the mean of three replicates ± standard deviation. Different letters indicates the significant differences among samples (*p*-value ≤ 0.05).

To prove that these strains can indeed cause the hydrolysis of protein in chickpeas during the fermentation process, we have tested the nitrogen content of the fermentation residue of 16 strains at the time point when the peptide yield was the highest during the fermentation process. It can be seen from the Figure 2 that the degree of hydrolysis calculated by this method is more than 60%, which is much higher than that produced by the enzymatic hydrolysis of chickpea protein by Rivero-Pino et al. (20). One possible reason for this difference is the different detection methods, and the other is that the preparation process used in this study is more efficient than other processes. It is reported that a high degree of hydrolysis may produce high biological activity, so we judge that chickpea peptides produced by these strains may have strong biological activity (20).

### Sodium dodecyl sulfate-polyacrylamide gel electrophoresis

**Figure 3:**
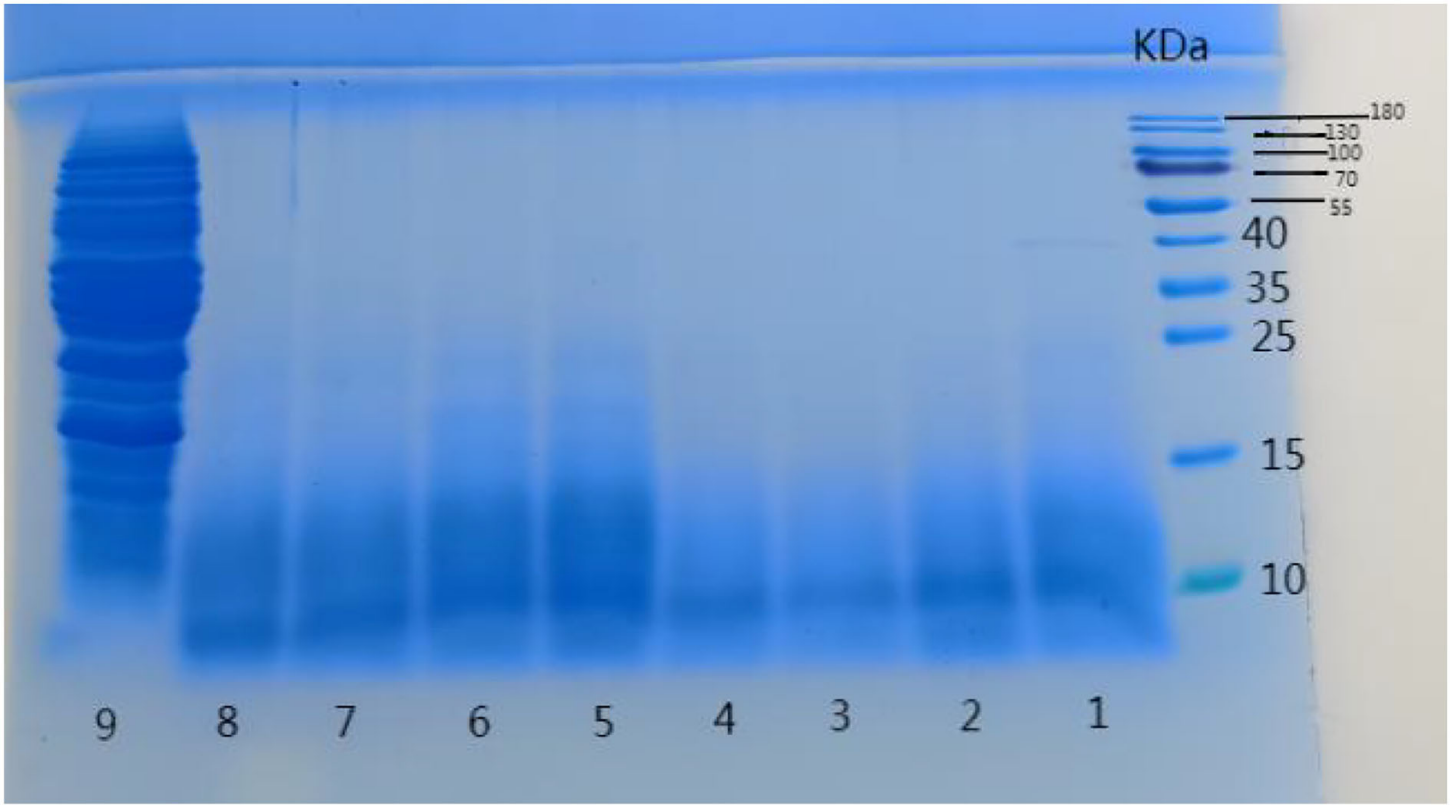
Polyacrylamide gel electrophoresis with sodium dodecyl sulfate for the fermentation of peptides from 8 strains, different swimming lanes indicate chickpea peptides fermented by different strains. Lane 1: *Bifidobacterium species*, lane 2: *Lactobacillus acidophilus*, lane 3: *Lactobacillus rhamnosus*, lane 4: *Lactobacillus paracasei*, lane 5: *Lactobacillus fermenti*, lane 6: *Lacillus subtilis*, lane 7: *Lactobacillus bulgaricus*, lane 8: *Lactobacillus casei*, and lane 9: Raw material.

Chickpea peptides fermented by 8 strains selected in Figure 1 were analyzed by sodium dodecyl sulfate polyacrylamide gel electrophoresis (SDS-PAGE). It can be seen from Figure 3 that the electrophoresis results of eight strains show that the molecular weight of peptide liquid obtained after fermentation is mostly below 10 kD, and basically below 25 kD. This result is basically consistent with the results of Acevedo et al. hydrolyzed chickpea protein with bromelain (4). Compared with the raw materials, the eight strains played a good role in the decomposition of raw protein, but the fermentation effect of the strains in the first four lanes was significantly better than that of the last four. In contrast, the molecular weight of chickpea peptide in the first four lanes was almost all distributed below 15 KD, and that in the last four lanes was almost all distributed below 25 kD. One possible reason is that the first four strains are more suitable for growing in raw materials and decomposing raw protein. Therefore, it can be preliminarily concluded that *Bifidobacterium species, Lactobacillus acidophilus, Lactobacillus rhamnosus*, and *Lactobacillus paracasei* have significantly stronger protein decomposition ability than the other four strains. Finally, we screened the chickpea peptides fermented by four strains with better protein decomposition effects for further analysis and research.

### Analysis of peptide molecular weight distribution by high-performance liquid chromatography

We have analyzed the molecular weight distribution of chickpea peptide, which was fermented by four strains with the best results of sodium dodecyl sulfate polyacrylamide gel electrophoresis (SDS-PAGE)

**Figure 4:**
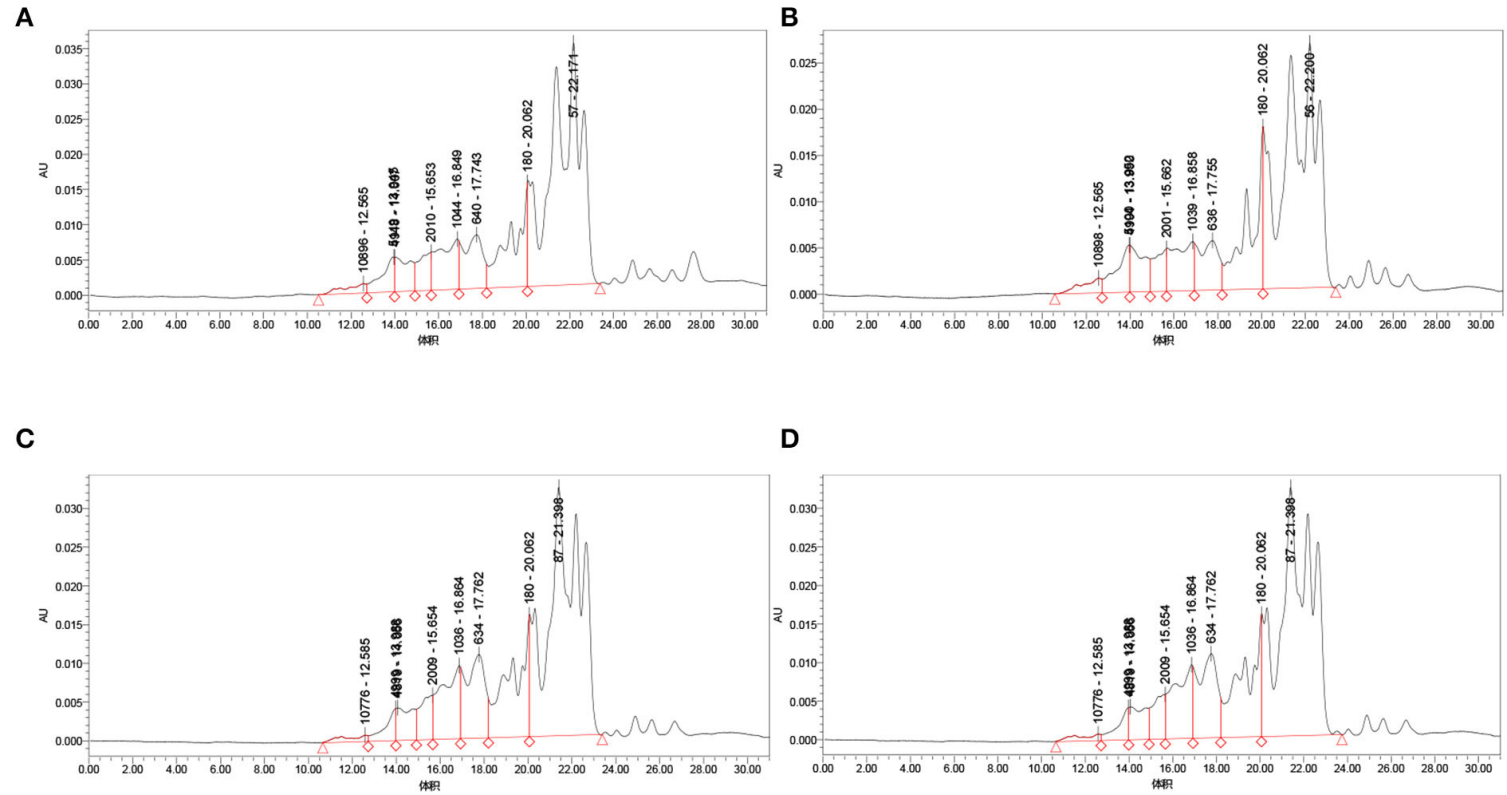
Molecular weight distribution of fermentation peptides of four strains. **(A)** Molecular weight distribution of *Bifidobacterium species* fermentation peptide; **(B)** *Lactobacillus acidophilus* molecular weight distribution of fermentation peptide; **(C)** *Lactobacillus rhamnosus* fermentation peptide; and **(D)** *Lactobacillus paracasei* fermentation peptide.

As shown in Figure 4 and Table 1, the proportion of chickpea peptide 3–10 kD and >10 kD obtained by *Lactobacillus acidophilu*s fermentation is significantly higher than that of the other three strains, and the proportion of chickpea peptide <1 kD is significantly lower than that of the other three strains. The molecular weight distribution of fermented chickpea peptide is very similar, and the contents of 3–10 kD and more than 10 kD are less than those of the other two strains, and the contents of <1 and 1–3 kD are significantly higher than those of the other two strains. However, the molecular weight distribution of chickpea peptide fermented by *Lactobacillus rhamnosus* and *Lactobacillus paracasei* is very similar, both of whose 3–10 kD components and components >10 kD were less than that of the other two strains as well as the proportion of components <1 and 1–3 kD components were significantly higher than that of the other two strains. In general, the molecular weight of the peptides fermented by the four strains is mostly distributed below 3 kD, which indicates that the fermentation is so sufficient that most of the proteins are decomposed into small peptides or amino acids. However, the molecular weight distribution of the peptide fermented by four different strains was not very consistent. The possible reason for this difference is that different strains produce different protease cleavage sites. Studies have shown that small peptides composed of 3–20 amino acids have stronger biological activity (21). Most of the four fermented peptides in the above figure are small molecular peptides <10 kD, so it is speculated that these peptides may have certain biological activity (22–24).

**Table 1:** Molecular weight distribution of fermentation peptides of four strains.

		**Peak area ratio/%**		
**Name**	** <1000KD**	**1KD~3KD**	**3KD~10KD**	**>10KD**
*Bifidobacterium species*	78.25	12.02	8.06	1.67
*Lactobacillus acidophilus*	77.29	11.22	9.36	2.13
*Lactobacillus rhamnosus*	79.53	13.23	6.16	1.08
*Lactobacillus paracasei*	79.5	13.21	6.19	1.1

### Analysis of the amino acid content

Table 2 shows that the contents of glutamate (0.813650 mg/ml), histidine (0.144165 mg/ml), glycine (0.206894 mg/ml), threonine (0.206894 mg/ml), lysine (0.122345 mg/ml), and proline (0.186931 mg/ml) in chickpea fermented by *Lactobacillus acidophilus* are significantly higher than those of the other three strains. The serine content (0.165638 mg/ml) and arginine content (0.323349 mg/ml) of chickpea fermented by *Lactobacillus rhamnosus* fermentation were significantly higher than those of the other three strains. The contents of cystine (0.0965911 mg/ml), valine (0.185441 mg/ml), methionine (0.0856057 mg/ml), and lysine (0.176895 mg/ml) in chickpea fermented by *Lactobacillus paracasei* were significantly higher than those of the other three strains. The total amount of hydrophobic amino acids from chickpea fermented by *Lactobacillus acidophilus* was 0.8774369 mg/ml, and the total amount of hydrophobic amino acids from chickpea fermented by *Bifidobacterium species* was 0.7655171 mg/ml. The total amount of hydrophobic amino acids from chickpea fermented by *Lactobacillus rhamnosus* was 0.8332841 mg/ml, and the total amount of hydrophobic amino acids from chickpea fermented by *Lactobacillus paracasei* was 0.8762248 mg/ml. The reason for this difference may be that different strains produce different protease cutting methods and sites (25), resulting in the difference of amino acid content in fermentation products. Different amino acid content will make the product have different biological activities, so we think it is an important reason for these peptides to have different α-glycosidase inhibitory activities (26, 27).

**Table 2:** Amino acid contents of fermentation peptides of four strains.

**Name/(mg/mL)**	***Bifidobacterium species***	***Lactobacillus acidophilus***	***Lactobacillus rhamnosus***	***Lactobacillus paracasei***
Aspartic acid	0.287192	0.328407	0.222772	0.347873
Glutamate	0.519431	0.813650	0.536336	0.611797
Serine	0.102053	0.135023	0.165638	0.109781
Histidine	0.0945721	0.144165	0.0956853	0.0950773
Glycine	0.174845	0.206894	0.188335	0.190026
Threonine	0.105396	0.150318	0.0974209	0.106289
Arginine	0.182791	0.220769	0.323349	0.220136
Alanine	0.149717	0.168545	0.158194	0.168571
Tyrosine	0.0573061	0.0606248	0.0576242	0.0466678
Cysteine	0.0531949	0.0304334	0.0351882	0.0965911
Valine	0.128106	0.144507	0.140511	0.185441
Methionine	0.0481661	0.0635783	0.0655561	0.0856057
Phenylalanine	0.0727520	0.0834904	0.0856852	0.0813175
Isoleucine	0.0885850	0.0964812	0.0992848	0.0968126
Leucine	0.123450	0.133904	0.135581	0.133101
Lysine	0.153433	0.122345	0.158123	0.176895
Proline	0.154741	0.186931	0.148472	0.125376

### Inhibition rate of α-glucosidase by chickpea peptides fermented by four strains

**Figure 5:**
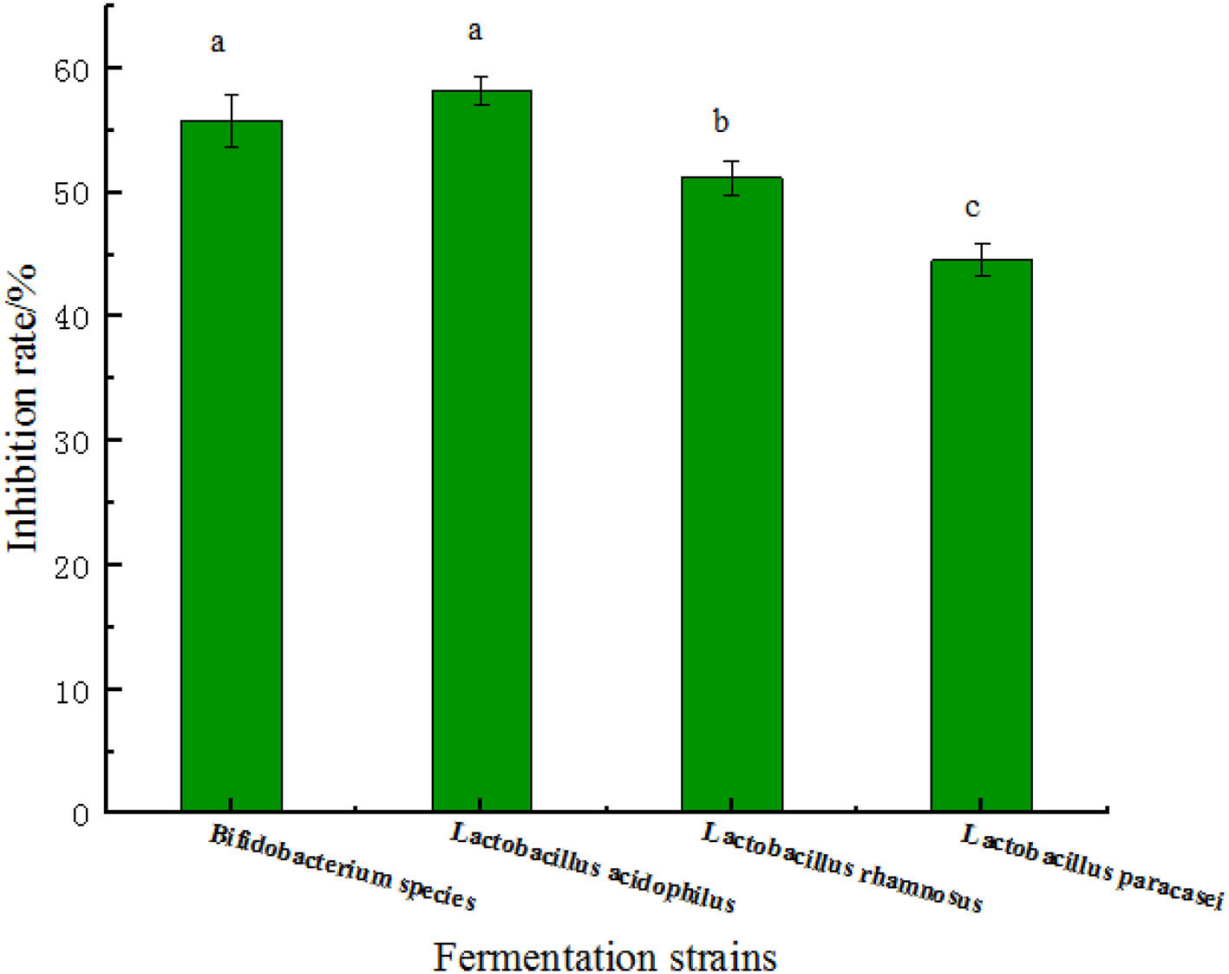
The rate of α-glucosidase inhibition by four fermentation peptides of strains at the concentration of 5.0 mg/ml. Values are presented as the mean of three replicates ± standard deviation. Different letters denotes significant differences among samples (*p*-value ≤ 0.05).

A-glucosidase inhibitors reduce the digestion of carbon and water through competitive inhibition, resulting in delayed glucose absorption and reduced hyperglycemia without insulin secretion (28). It can be seen from the Figure 5 that the α-glycosidase inhibition rate of the chickpea peptide fermented by *Bifidobacterium species* and *Lactobacillus acidophilus* was significantly higher than that of *Lactobacillus rhamnosus* and *Lactobacillus paracasei* (at the level of *p* < 0.05), and the inhibition rate of the chickpea peptide fermented by *Lactobacillus acidophilus* was the best, with 58.22 ± 1.10% at the concentration of 5.0 mg/ml. One of the possible reasons is that the content of hydrophobic amino acids in the fermented peptide solution is the highest (0.8774369 mg/ml). Compared with the peptide extracted from *Paeonia ostii* cake by Qiao et al., the inhibitory effect of chickpea peptide in this study to α- glucosidase is quite obvious (29). It is pointed out in the literature that the higher the content of hydrophobic amino acids, the higher positive effect of the inhibition to α-glycosidase. Abbasi, Moslehishad, and Salami found that leucine, glutamic acid, arginine, glycine, and alanine significantly affect the effect of quinoa peptide on α-glucosidase inhibitory (30). Hydrophobic aliphatic amino acids, such as glycine, leucine, and alanine can promote the anti-diabetes ability of protein hydrolysates and are potential therapeutic agents for hypertension and diabetes (31). In addition, the presence of basic (arginine) and sulfur-containing amino acid residues and their length can be enhanced, which in turn increases the potency of glucosidase inhibitory activity. Pramai et al. studied the extract of germinated rice and found that glutamate and leucine may be one of the key metabolites helpful to improve glucosidase inhibitory activity (32). In addition, the inhibitory activity of α-glucosidase is related to the arrangement of amino acids and the structure of peptides (33).

Many peptides with α-glycosidase inhibitory activity can be extracted from plant-derived proteins. Wang et al. (34) extracted an α-glucosidase inhibitory peptide LPLLR from walnut protein, and calculated its IC50 to be about 1.222 mg/ml (34). Hu et al. (35) isolated the peptide LRSELAAWSR from a spirulina, and its inhibitory activity against α-glucosidase was IC50 = 134.2 μg/ml. Among peptides from other sources, Ujiroghene et al. (31) identified novel hypoglycemic peptides from quinoa yogurt drinks.

### The α-glucosidase inhibition rate of different molecular weight fractions

**Figure 6:**
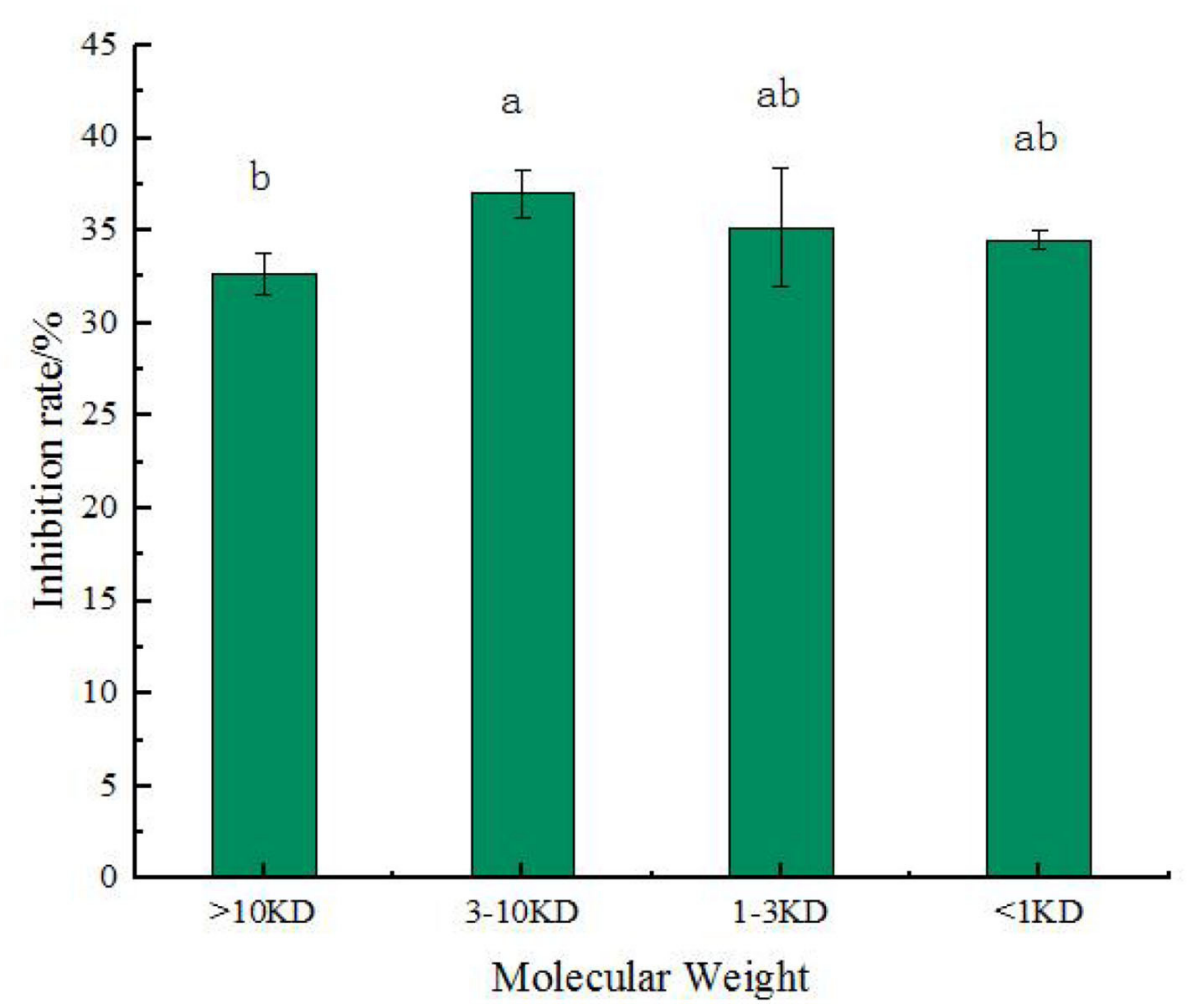
The rate of α-glucosidase inhibition by four molecular weight in the chickpea peptide fermented by *Lactobacillus acidophilus*. Values are presented as the mean of three replicates ± standard deviation. Different letters denotes significant differences among samples (*p*-value ≤ 0.05).

Figure 6 shows that chickpea peptide with the highest α-glycosidase inhibition rate, which was fermented by *Lactobacillus acidophilus*, was intercepted by ultrafiltration centrifuge tubes = 1, 3, and 10 kD. Four components were obtained, which included molecular weight <1 kD, 1–3 kD, 3–10 kD, and more than 10 kD. These four components were tested for α-glucosidase inhibition rate at the concentration of 2.0 mg/ml. From the results, we can see that the inhibitory effect of components with molecular weight >10 kD is significantly less than that of components with the molecular weight of 3–10 kD (*p* < 0.05), and the inhibitory effect of components with molecular weight <1 and 1–3 kD is not significantly different from that of components with molecular weight >10 or 3–10 kD (*p* < 0.05). It shows that in chickpea fermented peptides, when the molecular weight is <10 kD, the influence of the molecular weight of the peptide on the inhibitory activity is not very prominent. When the molecular weight is 3–10 kD, the inhibitory effect is the best, and the inhibitory rate is 37 ± 1.32%. Previous studies have shown that some plant protein peptides can inhibit α-glucosidase activity, such as the inhibitory activity of yellow pea peptide samples (53.35 ± 2.78% at 20 mg/ml) (25). Feng et al. (36) reported that the inhibition percentage of 2 mg/ml camellia seed peptide obtained by enzymatic hydrolysis with different proteases ranged from 9.88 to 20.44%, and the highest inhibition activity of wheat gluten subunit hydrolysate was 18.4 ± 0.7% (37). Compared with these peptides, chickpea fermented peptides showed excellent inhibitory activity of the α-glucosidase, especially when the molecular weight is 3–10 kD.

## Conclusion

In this study, eight strains with the best peptide-producing effect were screened out from the existing 16 strains by using the yield of peptide as the index, and then the four strains with the best proteolysis effect were screened out from the eight strains by SDS-PAGE. The molecular weight distribution and amino acid content of the four selected strains were detected by HPLC, and the inhibitory effect of α-glucosidase was detected. It was found that the molecular weight of most of the chickpea peptides fermented by these four strains was below 10 kD, and the α-glucosidase was inhibited to a certain extent by these peptides. By studying the inhibitory activities of different molecular weight components of chickpea peptide fermented by *Lactobacillus acidophilus*, which has the highest inhibition rate of α-glycosidase, it was found that molecular weight has little influence on the inhibitory activity in the range of 0–10 kD. The medium molecular weight chickpea peptide (3–10 kD) had the best inhibitory effect on α-glucosidase. Future work may include the identification, isolation, and α-glucosidase inhibitory activity test of each major component in chickpea fermented peptide solution, and the use of cell and animal models to reveal the antidiabetic mechanism of each component in chickpea fermented peptide.

## Data availability statement

The original contributions presented in the study are included in the article/supplementary material, further inquiries can be directed to the corresponding authors.

## Author contributions

XM, XF, DW, and XLi conceived and designed the study. XW, JY, CQ, and XLiu collected the data. JY, CQ, DW, and XLi performed data analysis. XM, XF, LW, RA, and GP made the figures. XM wrote the manuscript. All authors reviewed, approved the final version of the manuscript, and agreed to be accountable for the content of the work.

## Funding

This work was funded by Key R&D and Design Project of Xinjiang Uygur Autonomous Region, No.2021B02001-3-1.

## Conflict of interest

Authors XLi, XW, and RA were employed by Xinjiang Arman Food Group Co. Ltd. Author GP was employed by Shenxin Science and Technology Cooperation Base Co. Ltd. The remaining authors declare that the research was conducted in the absence of any commercial or financial relationships that could be construed as a potential conflict of interest.

## Publisher's note

All claims expressed in this article are solely those of the authors and do not necessarily represent those of their affiliated organizations, or those of the publisher, the editors and the reviewers. Any product that may be evaluated in this article, or claim that may be made by its manufacturer, is not guaranteed or endorsed by the publisher.
